# Characterization of Volatile Compounds and Flavor in Spirits of Old Apple and Pear Cultivars from the Balkan Region

**DOI:** 10.3390/foods10061258

**Published:** 2021-06-01

**Authors:** Nermina Spaho, Fuad Gaši, Erich Leitner, Milenko Blesić, Asima Akagić, Sanja Oručević Žuljević, Mirsad Kurtović, Davorka Đukić Ratković, Mirela Smajić Murtić, Milica Fotirić Akšić, Mekjell Meland

**Affiliations:** 1 Faculty of Agriculture and Food Sciences, University of Sarajevo, 71000 Sarajevo, Bosnia and Herzegovina; n.spaho@ppf.unsa.ba (N.S.); f.gasi@ppf.unsa.ba (F.G.); m.blesic@ppf.unsa.ba (M.B.); a.akagic@ppf.unsa.ba (A.A.); s.orucevic-zuljevic@ppf.unsa.ba (S.O.Ž.); m.kurtovic@ppf.unsa.ba (M.K.); m.smajic-murtic@ppf.unsa.ba (M.S.M.); 2 Institute of Analytical Chemistry and Food Chemistry (6450), Graz University of Technology, 8010 Graz, Austria; erich.leitner@tugraz.at; 3 Disttllery BMB Delta, Jablanica, 173, 78405 Gradiška, Bosnia and Herzegovina; davorkaratkovic@yahoo.com; 4 Faculty of Agriculture, University of Belgrade, Nemanjina 6, 11080 Belgrade, Serbia; fotiric@agrif.bg.ac.rs; 5 Norwegian Institute of Bioeconomy Research, NIBIO Ullensvang, Ullensvangvegen 1003, N-5781 Lofthus, Norway

**Keywords:** old apples, old pears, fruit spirits, volatile aroma compounds

## Abstract

This study was conducted with the aim of developing fruit spirits by utilizing old (autochthonous) apple and pear cultivars that can be attractive to both consumers and producers. Consumers of spirits could enjoy the unique flavor, and producers could gain an opportunity for brand development. In total, eight old apple cultivars (Sarija, Žuja, Samoniklica, Prijedorska zelenika, Bobovec, Masnjača, Lijepocvjetka, and Šarenika) and three pear cultivars (Budaljača, Krakača, and Kalićanka) from Bosnia and Herzegovina were used for the spirits production and for characterizing the flavor of distillates. Golden Delicious was used as a representative of commercial apple cultivar. The aroma profile was conducted through the identification of minor volatile organic compounds (VOCs) and the sensory perception of spirits. Analysis of the VOCs was performed by gas chromatography mass spectroscopy (GC/MS) techniques after enrichment via solid-phase microextraction (SPME). Sensory evaluation was performed by 12 trained panelists. Overall, 35 minor volatile compounds were found in spirits: 13 esters, 7 alcohols, 6 acids, 5 terpenes, and 4 aldehydes. Significant differences were detected in the distribution and quantity of the VOCs, which were fruit cultivar-dependent. Spirits made from Šarenika apple cultivar showed the largest amount of all acids, especially short- and medium-chain fatty acids; however, this richness was not correlated with pleasant sensory attributes. Spirits obtained from Prijedorska zelenika and Masnjača apple cultivars had the best sensory attributes. Budeljača and Krakača pears are promising cultivars as flavoring in spirits production.

## 1. Introduction

Old fruit cultivars from Bosnia and Herzegovina (B&H) represent an interesting genetic resource and require to be protected and adequately used [1]. Old fruit cultivars present in B&H have previously been investigated in terms of their genetic diversity [2,3], morphogenic variability [4], biochemical composition of their fruits [2,5], polyphenolic profile [6], as well as sensory properties [1]. However, few studies [7,8] have considered the acceptability of old apple and pear in fruit spirits production. Increased consumption of fruit in B&H is evident, but it is not as large as hoped [9]. Studies conducted by Iccariano et al. [10] and Akagić et al. [11] showed that juice from fruit from old apple cultivar is characterized both by health-promoting properties and distinguishing sensory attributes. In the last decade, there have been many studies that evaluated either the fruits from old apple cultivars [12,13,14] or the juice obtained from them [15].

Fruit spirits are popular alcohol beverages due to their unique flavor. Very often, specific spirits represent the national drink of the country and they are regarded through a lens of tradition or gastronomic heritage [16]. Fruit spirits are characterized by intensive and typical fruit flavor. They are products of distillations, a technique that concentrates volatile organic compounds (VOCs) from fermented fruit mash, juice, or vine [16]. Distillates are ultimately a mix of a huge number of VOCs that determine the sensory profile of the distillate. These compounds can be used to classify beverages by type and raw material [17,18] and give a unique odorant characteristic to a drink. The flavor of fruit distillates originates from four sources [16]: fruits (primary flavor), fermentation (secondary flavor), distillations (tertiary flavor), and maturation (quaternary flavor). The most common VOCs in distillates are major fermentative aroma compounds, which are produced during common and specific fruit fermentation. Investigations of major volatile compounds in apple and other fruit spirits have been conducted by numerous studies [18,19,20,21,22], while minor volatile compounds have been studied far less [23,24,25]. Minor volatile compounds are present in apple juice or cider in a very low concentration but have a significant influence on the overall sensory attribute [26,27,28,29].

This study focused on the determination of minor volatile compounds in fruit spirits for characterizing specific flavors of distillates obtained from old apple and pear cultivars.

## 2. Materials and Methods

### 2.1. Plant Material

Based on the results of a previous study on sensory evaluation of Bosnian and Herzegovinian old apples and pears [1], 8 apple and 3 pear cultivars were selected for this study. The old apple cultivars used for the production of spirits were the following: Sarija, Žuja, Samoniklica, Prijedorska zelenika (P. zelenika), Bobovec, Masnjača, Lijepocvjetka, and Šarenika. The cultivar Golden Delicious (G. Delicious) also used as a representative of commercial apple cultivars, since it yields spirits with floral sensory attributes [25,30]. Budaljača, Krakača, and Kanjiška were the old pear cultivars used in the study. All fruits from the selected apple and pear cultivars were harvested within ex situ collection “Srebrenik”, located in Northeast Bosnia, altitude 166 m a.s.l. and GPS coordinates 44°45′45″ N 18°29′49″ E. The climate is moderately continental, and the soil is alluvial–deluvial. Standard commercial practice was used in orchard management. Fruits were picked at commercial harvest stage. Harvest time varied depending on fruit ripening time from the end of July to the middle of October in 2014.

### 2.2. Fruit Mash Preparation and Spirits Production

Immediately after harvest, the fruits were successively transported to pilot plant, located at the Faculty of Agriculture and Food Science, University of Sarajevo. Upon delivery, each cultivar was immediately crushed by the rotating rollers with stainless steel teeth, and seeds were not removed. The main chemical parameters (acidity and extract content of fruit mash) were measured upon delivery and milling on standard apple mill. The results are presented in Table 1. The mash (80 kg per fruit) was corrected to approximately pH 3.0 with the addition of a necessary amount of 1:10 diluted solution of sulphuric acid in accordance with base pH of the fruit. Fermentations were performed using commercial *Saccharomyces cerevisiae* (Uvaferm, Danstar Ferment AG, Syddanmark, Denmark) in dozage according to the manufacturer’s instructions (20 g/hL) Fermentations were conducted in four closed tanks of 20 L, for each fruit cultivar. Fermentation were carried out for approximately 10 days in summer time at 23 ± 3 °C and two weeks in the fall at 19 ± 2 °C. The rate of alcohol fermentation was monitored daily by measuring residual sugars by hand refractometer. The fermentation was considered completed when the extract was below 3.5 °Brix.

Immediately upon completion of fermentation, fermented mash was distilled using an alembic copper pot still. Two-stage distillation was performed. In the first distillation, a raw distillate (low spirits) was obtained and it represented the average of 2 pot still distillations. There was no fraction cutting during the first distillation, since the aim was to exhaust alcohol from the fermented mash. The alcohol strength of low spirits was around 18–25% (*v*/*v*) depending on the sugar content in the fruit. The second distillation was run with separation into three fractions: head, heart, and tail. The same head (1.2% by volume) and tail cuts (until the alcohol decreased to 40% *v*/*v*) were carried out. The final alcoholic degree in the heart cut ranged between 58.8% and 62.3% *v*/*v*, depending on how rich in alcohol the low spirits had been. All the spirts samples were kept in dark bottles at −18 °C until analyzed. The described experiment was performed in duplicates.

### 2.3. Sample Preparation and GC-MS Analysis

A sample of 10 µL of each distillate was transferred by micro capillaries (Hirschmann ringcaps, Eberstadt, Germany) into 20 mL headspace vials. After the addition of glass-coated magnetic stir bars, magnetic crimp caps with a polytetrafluoroethylene (PTFE)-lined silicone septum were used to close the vials. The volatile fraction was enriched on a 2 cm stable flex 50/30 µm Divinylbenzene/Carboxen/PDMS SPME fiber (Supelco, Bellefonte, PA, USA) for 20 min at 60 °C. Desorption took place directly in the heated injection board of a GC-MS system at 270 °C with a 0.75 mm inner diameter SPME liner.

For the separation of the volatiles, a Shimadzu QP-2020 GC MS system (Shimadzu, Duisburg, Germany) with a single quadrupole mass-selective detector was used in scan mode. A 30 m Restek Rxi5MS (0.25 mm inner diameter and 1 µm film thickness, Restek, Bellefonte, PA, USA) with the following temperature program was used: 20 °C (1 min) with a ramp of 8 °C/min to 270 °C (1 min) with helium as carrier gas in constant flow mode with a linear velocity of 35 cm/s.

Data were acquired in scan mode with a scan rate of 3.3 scans/s from 35 to 350 amu. The detector multiplier voltage was set 50 V below tuning voltage to prevent detector saturation. Interface was set to 280 °C, and ion source temperature was set to 200 °C. All samples were analyzed in duplicate.

All chemicals were obtained from Sigma-Aldrich (Steinheim, Germany) and Fluka Chemie GmbH (Buchs, Switzerland). All reagents used were of analytical grade of purity.

### 2.4. Sensory Analysis

A single sample for sensory analysis was made as an average of two repetitions. Fifteen days before the evaluation, samples were diluted with water to an ethanol content of 40% *v*/*v* and kept in a fridge at 4 °C.

Sensory evaluation of twelve spirts (nine apple and three pear cultivars) was performed by a panel consisting of 12 trained assessors, 7 men and 5 women (22–55 years of age). Assessors were recruited from the staff of the Faculty of Agriculture and Food Science at the University of Sarajevo. In the first part of the training, a list of sensory attributes and corresponding reference standards, according to Qin et al. [31], were presented and discussed by the assessors during two sessions. Following this, assessors were trained in the evaluation of apple and pear spirits and in the use of spirits attributes according to procedures described by Rodrıguez Madrera et al. [32] and Caldeira et al. [33]. A total of six training sessions were held. The following ten attributes were used for sensory profiling of the spirits’ flavor: odor intensity, typically, fruity, floral, herbaceous, spicy, chemical, fatty/cheesy, pungent, and after taste. Flavor attributes were rated using a five-point scale system: 1—very weak, 2—weak, 3—moderate, 4—strong, and 5—very strong. The samples were served in tasting glasses at room temperature (20 °C). The glasses were coded with a three-digit number from a table of random numbers. Apple and pear spirits were evaluated separately. Each assessor evaluated three randomly distributed samples in one round and in total four rounds in order to evaluate all samples. Presentation of the samples was carried out by the random balance order, avoiding first-order carryover effects. The results are presented as an average grade of assessors, where the assessors performed repetitions.

### 2.5. Statistical Analyses

The results of chemical analysis are presented as mean relative amount, expressed as the peak areas just from extracted ions after proper identification of the substances with coefficient of variation. The results of sensory analysis were subjected to one-way analysis of variance (ANOVA) to evaluate whether significant differences existed between the sensory attributes of apple and pear distillates according to cultivar. The established differences of mean values were tested by Tukey’s test. A multivariate analysis, namely, principal component analysis (PCA), was performed to investigate the relationships between the VOCs and sensory attributes of the 12 spirits obtained from eight apple and three pear cultivars. Data were analyzed by the statistical package StatBox 6.7 (Grimmersoft, Paris, France).

## 3. Results and Discussion

### 3.1. Analysis of the Volatile Organic Compounds

**Figure 1:**
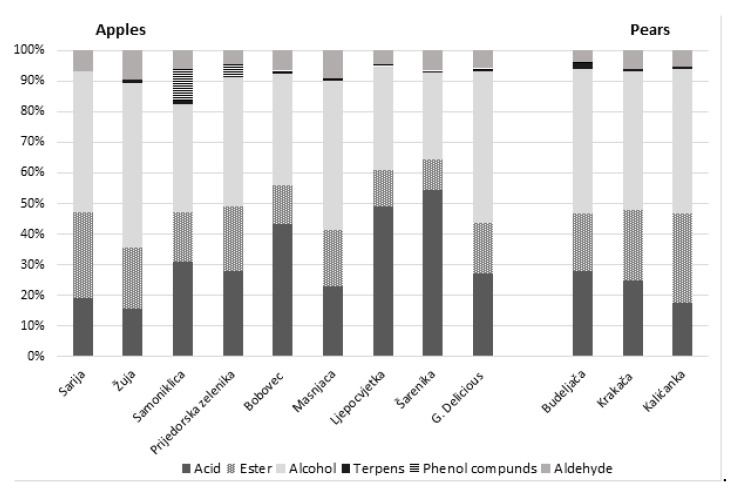
Relative distribution (%) of main compound classes in spirits from old apple and pear. cultivars from B&H.

VOCs were determined by GC-MS after enrichment of the volatiles by headspace solid-phase microextraction (HS-SPME-GC-MS). In total, 36 VOCs were identified, and these are presented as average peak areas in Table 2, according to the following chemical classes: acids, esters, alcohols, terpenes, and aldehydes. The relative distribution of the main compound classes, detected among the apple and pear spirits, is presented in Figure 1. There were obvious and large differences in the average percentage shares for the different chemical classes among the analyzed fruit spirits.

Among all the chemical classes detected in the volatile content of spirits, esters were present in the highest number (13), followed by alcohols (7), acids (6), terpenes (5) and aldehydes (4). Esters and higher alcohols are qualitatively and quantitatively predominated macro-constituent of fruit spirits [7,34,35]. However, even among micro-constituent, esters and higher alcohols had large share in the overall VOCs profile. Contrary, in spirts made from apple cultivars Šarenika, Ljepocvjetka and Bobovec, acids had the highest share of VOCs (Figure 1).

Since free volatile acids are converted into esters, in the presence of ethanol [17], their substantially share in the VOC profile of these samples was surprising. Higher content of fatty acids ethyl esters compared to free fatty acids has been reported in cider [27], Calvados [36], sugarcane spirits [37], Mouro distillates [38], and equable content of free fatty acids and its correspondent esters in some cider distillate [39]. However, the results of other studies showed a higher level of free fatty acid than ethyl esters of short and medium-chain fatty acid in the different type of alcoholic beverages [40,41,42]. Minor prevalence of the VOCs belonging to aldehyde, phenol and terpene classes, detected in this study, was expected.

### 3.2. Volatile Organic Compounds in Apple Spirits

Detectable acids in apple spirits consisted of three short-chain fatty acids (Butanoic acid; Butanoic acid, 3-methyl-; Butanoic acid, 2-methy) and three medium-chain fatty acids (Hexanoic acid; Octanoic acid; Decanoic acid <n->). They were identified in varying amounts. Fatty acids with short and middle chains are a natural component of fruit [43,44] and are also formed by the activities of bacterium and yeasts during the fermentation process. Among the detected acids, in the examined samples, the most abundant was butanoic acid, 3-methyl, which was presented in a very high level in Šarenika, Ljepocvjetka, and Samoniklica apple cultivars spirits, respectively (Table 2). This agrees with the previous study of Greek fruit distillate ‘‘Koumaro’ [40], where, based upon the relative peak area percentage, octanoic acid was the predominant acid in the majority of the produced apple spirits. The same was determined in our study regarding Prijedorska zelenika, Sarija, Masnjača, and Golden Delicious apple distillates. Aside from the acetic acid, which represents the major acid in alcoholic drink, octanoic acid is the predominant acid in apple fermented juice [26]. Hexanoic acid was consistently present in large amounts in all apple samples, in this study.

Short-chain free fatty acids have unpleasant odors, similar to those of rancid butter and putrid cheese, and are not desirable in a high amount in alcoholic beverages [16]. High levels of these acids could be an indicator of poor mash quality. Medium-chain free fatty acids have a lesser flavor effect on distillates [38,45]. The odor of the mentioned octanoic acid is described as similar to that of butter or almond, the hexanoic acid contributes to leafy, woody, varnish odor, while decanoic acid contributes to a caramel odor [42].

In this experiment, the class of esters was represented with five esters of short-chain fatty acids, six esters of medium-chain fatty acids, and two esters of long-chain fatty acids. This class was qualitatively the most numerous, but it surprisingly showed a small share among apple spirits’ VOCs profile (Figure 1). Rather, high variations of esters were evident, with substantially higher levels detected in apple spirits of Sarija and Prijedorska zelenika cultivars and the lowest levels in spirits of G. Delicious and Bobovec cultivars (Table 2). There are numerous factors that influence the ester formation. Ethyl ester can arise from the raw material [43,44], they are produced during fermentation [34], as well as during distillations, when the heat releases a significant amount of ethyl ester of fatty acids from the yeast cell previously bound after the fermentation [38].

The fatty acid esters contribute to a fruity and flowery aroma, but their contribution to the aroma profile of spirits is strongly influenced by their concentration [16]. The most abundant ester in all analyzed apple spirits was ethyl lactate (Table 2). It was the most prominent in the apple spirts of Sarija and the pear spirits of Kalićanka and was least in the spirits made from G. Delicious. Because of its high concentration in fruit and grape spirits, it is sometimes considered as a major VOC [31,46]. Ethyl lactate has a positive effect on the distillate aroma only when it is present at low concentration [47]. It stabilizes the distillate’s flavor and softens its harsh character [48]. A very high concentration of ethyl lactate could indicate that bacterial spoilage occurred during fermentation. Aside from ethyl lactate, a high level of ethyl octanoate, ethyl dodecanoate, butanedioic acid diethyl ester (diethyl butanedioate or diethyl succinate), and ethyl hexanoate was detected. The other esters, such as isobutyl acetate, ethyl isovalerate, isoamyl acetate, 2-methylbutyl acetate, methoxy acetic acid, 3-methylbutyl ester, ethyl decanoate, ethyl tetradecanoate, and ethyl hexadecanoate, were present in smaller quantities. Similar results, obtained on spirits from Sardinian apple varieties, were reported by Veresini et al. [7], with the exception of diethyl butanedioate content, which was not detected in their samples. On the other hand, in earlier studies [40,49,50], the high content of diethyl butanedioate has also been found in Ojuro, apple, and blackberry distillates. A moderate quantity of butanedioic acid diethyl ester also belongs to fruity-apple-type odor. An increased quantity of butanedioic acid diethyl ester can be a consequence of malolactic fermentation that can cause an increase in the concentration of butanedioic acid diethyl esters resulting in a loss of fruitiness and aroma intensity [51]. Nevertheless, the high content of butanedioic acid diethyl ester in Šarenika and Prijedorska zelenika apple spirits was not accompanied by a high amount of ethyl lactate (product of malolactic fermentation). This means that the level of diethyl butanedionate probably was not linked to bacterial spoilage. The content of diethyl butanedionate managed to distinguish the Brasilian grapa marc samples from Italian grappa according to results reported by Serafim et al. [52]. In that sense, diethyl butanedionate could be used as a biomarker for differentiation of Prijedorska zelenika and Šarenika spirits from another apple spirits. An investigation by Schmutzer et al. [53] showed that the absence of diethyl butanedionate may suggest that alcohol drinks were counterfeited. Very high proportions of ethyl isovalerate were detected among Šarenika and Ljepocvjetka apple samples. This ester is an important odorant in apple and ciders [36]. The Šarenika cultivar had the highest level of ethyl hexanoate, an ester which supplies an aroma of fruit, described as aroma of apple peel [31].

In comparison to G. Delicious spirits, all apple spirits obtained from old cultivars were characterized with a much higher level of aromatic ethyl esters of short-chain fatty acids. However, it was observed that the ester content of G. Delicious spirts increases with fatty chain length (Table 2). Usually the shorter-chain fatty esters are associated more with fruity and floral aroma than the longer-chain fatty acid esters are. Sweet-fruity, banana, and pear-like aroma in freshly distillated cognac is directly associated with a concentration of 2- and 3-methylbutyl acetates. Among these, 2-methylbutyl acetate is especially linked with the overripe fruit aroma [31]. The high content of ethyl hexanoate and ethyl butanoate in spirit may also enhance this “fruity” note [54].

Quantitatively speaking, higher alcohols constitute the main group of aromas in distillates [32], which was also confirmed in this study. Six out of eight apple cultivar spirits had the highest share of alcohols in their VOCs profile (Figure 1). The major higher alcohols in apple distillates are amylic alcohols, isobutyland n-propanol, accounting for approximately more than 90% of all higher alcohols in apple distillates [21,55]. The most abundant minor alcohol was phenylethyl, then 1-hexanol, 1-octanol, and benzyl alcohol. Lower content was observed for the 1-decanol and 1-pentanol, while 1-dodecanol had the lowest presence (Table 2). A dominant concentration of phenylethyl alcohol in apple or cider distillates has been reported in previous studies, by several authors [32,55,56], while other authors detected the highest values for 1-hexanol in apple distillates [7,57].

In this study, the highest levels of total alcohols were detected in Šarenika cultivars spirit, while Samoniklica cultivars spirits had the lowest amount of total alcohol (Table 2). The overall share of phenylethyl alcohol, whose flavor is considered pleasant, floral, rose-like, was the highest in Masnjača cultivars spirits, while the highest share of 1-hexanol, which is associated with a grassy scent in distillates, was detected in Šarenika cultivars spirits. Among all analyzed apple spirits, Šarenika spirit was also characterized by the highest level of 1-octanol, alcohol with a penetrating aromatic odor. Rather great variations of benzyl alcohol were evident among the spirits produced from various cultivars, with substantially higher levels detected in Šarenika and Masnjača cultivars spirits. The presence of this aromatic alcohol is associated with old sponge or mold aroma [58].

The chemical class of terpene was represented with two compounds, *cis*-linalool oxide and *trans*-linalool oxide (furanoid), which give a peculiar aroma contribution to fruit spirits. Both linalool oxides are varietal compounds [56] and could be used as a biomarker for distinguishing the fruit varieties used. Terpenes originate from fruit raw material and are intensively formed during fruit processing that occurs at high temperatures and low pH [48]. In such conditions, the release of bound terpenes into free terpenes occurs. The concentration of terpenes has been used to attest the sensorial quality of wines, beers, and distilled beverages [51]. A higher share in VOCs of apple samples was detected for cis-linalool oxide compared to *trans*-linalool oxide (Table 2). Especially high levels of these two linalool oxides were found in Samoniklica apple spirits, as well as slightly lower levels among Šarenika and Žuja cultivar’s spirits. Those spirits could be differentiated from other apple spirits by the content of linalool oxides. Sarija and G. Delicious cultivars spirits were characterized by low content of linalool oxides.

Phenol compounds detected in spirit samples were phenol, 4-ethylphenol, 4-ethyl-2-methoxyphenol (4-ethylguaiacol), and eugenol. Volatile phenols, especially 4-ethylphenol and 4-ethylguaiacol, are responsible for olfactory defects. They are produced by the contaminant Brettanomyces yeasts from grape (fruit)-derived phenolic acids. Volatile phenols in low concentration can be considered as normal constituents and can even contribute to positive sensory attributes and the complexity of aroma by imparting aroma notes of spices, smoke, and leather [59]. Brettanomyces infection is especially wanted in production of Belgian Lambic beer [60]. Coldea et al. [43] stated that the content of volatile phenols 4-ethylphenol, 4-ethylguaiacol, and eugenol should only be present in moderate amounts when compared to the other compounds. Anton et al. [28] noticed that the consideration of volatile phenols as marker of sensory defect should be revised since in analyzed cider samples, they did not find any sensory defect despite a high content of 4-ethylphenol to 4-ethylguaiacol. Similar results have been reported by Ledauphin et al. [58]. However, those two phenols are considered as part of the structure of Calvados and cider aroma [27,58]. Among other compounds, 4-ethylphenol is considered as a skeleton compound in Hennessy XO [61]. The odor of 4-ethylphenol was described as animal, 4-ethylguaiacol and eugenol as floral, hyacinth, or clove-like [57]. In high concentrations, 4-ethylphenol and 4-ethylguaiacol are responsible for varnish, pharmacy, off-flavors, smelling mousy, horse sweat, or rancid cheesy aroma [59]. The presence of 4-ethylphenol and 4-ethylguaiacol in Calvados, distilled cider, and cider has been detected in previous studies [27,32,37].

Based on their relative peak area percentage, the phenol 4-ethylphenol was the most present phenol compound in all examined apple spirits, followed by eugenol (Table 2). In VOCs profiles of studied apple spirits, the proportion of phenol compounds comprised a very small overall percentage, below 2% with the exception of Samoniklica and Prijedorska zelenika cultivars spirits (Figure 1). Samoniklica cultivars spirit was characterized with substantially higher level of 4-ethylphenol, while Prijedorska zelenika had very high levels of eugenol (Table 2). The sprits made from G. Delicious apples showed a lower content of all detected phenol compounds in comparison with most of the spirits obtained from old apple cultivars. Only Žuja and Sarija cultivars spirits had a lower content of phenol compounds than that of G. Delicious spirits.

The class of aldehyde was represented with 3-furaldehyde, 5-methyl-furfural, benzaldehyde, and nonanal. Aldehydes accounted for less than 10% of the minor aromatic composition among the spirits produced from old apple and pear cultivars (Figure 1). Benzaldehyde had a dominant share in total aldehyde amount, followed by 3-furaldehyde, 5-methyl-furfural, and nonanal (Table 2). A moderate variation of aldehyde compounds was detected among apple spirits produced from different cultivars, with significant levels in Šarenika, as well as slightly lower levels in Masnjača spirits. In comparison to old apple spirits, G. Delicious spirits had the lowest share of aldehydes in VOCs profile. Benzaldehyde and nonanal originate from the raw material so they could be considered as a raw material biomarker. Benzaldehyde is formed by hydrolysis of amygdaline from seeds or stones. Benzaldehyde has a low threshold, 35 µg/L [51], and contributes a biter almond, marzipan, cherry flavor to spirits [16]. The highest level of benzaldehyde was detected in Šarenika and Masnjača apple spirits, probably due to the higher share of seeds or greater chorusing of seeds during the apple milling. Nonanal is also a particular varietal compound with floral, fruity, green, and woody aroma contribution to spirits [62]. Spirits obtained from G. Delicious had the highest share of nonanal. 3-Furaldehyde and 5-methyl-furfural were not detected in other fruit spirits. They are created in thermal reaction by dehydration of various pentose, methyl pentose, and methyl pentosans in the presence of acids. 5-Methylfurfural has a sweet, spicy, warm odor with a sweet, bready, maple-like, caramel-like flavor [63].

### 3.3. Analysis of the Volatile Organic Compounds in Pear Spririts

In regards to pear spirits, there were notable differences among the investigated pear cultivars. Pear spirits in general possessed lower amounts of VOCs in comparison with most spirits obtained from old apple cultivars. Budaljača pear spirits contained the highest amount of hexanoic acid, while Krakača and Kalićanka pear spirits possessed an abundance of octanoic acid (Table 2). The highest share of acids within volatile profiles was noted in Budaljača pear spirits and then the spirits produced from Krakača fruit, while the smallest share of acids was detected in Kalićanka pear spirits. However, the exact opposite order was registered in regards to esters (Figure 1). The most abundant ester in pear spirits was ethyl lactate. Aside from ethyl lactate, the dominant esters in the analyzed pear samples were ethyl esters, including middle-chain fatty acids octanoic, decanoic, and hexanoic (Table 2). Similar results have been reported on spirits from Spain commercial pears varieties in two previous studies [8,64], as well as for the Williams pear spirits [65]. The mentioned esters are responsible for the fruity and floral flavor of the spirits. The overall share of esters in VOCS is dominant in Kalićanka cultivars spirits. This is a consequence of a substantially higher content of butanedioic acid, diethyl ester (diethyl succinate) and lactate ester found in Kalićanka pear spirits in comparison with the samples obtained from the two other cultivars. However, since the concentration of both esters is generally increased by bacterial spoilage, the level of these esters is linked more to the fermentation process than with any varietal properties.

The overall share of alcohols in VOCs was dominant and quite equal across all analyzed pear spirits (Figure 1). These high proportions of alcohol are not in accordance with reports by other authors, who noted that either esters or acids had the highest overall share within pear spirits’ VOCs profiles [8,17]. A comparison of values detected for individual alcohols indicates a large diversity among the analyzed pears spirits samples (Table 2). The impact of pear cultivar on the content of the seven individual alcohols, detected in this study, was previously reported by Arrieta-Garey et al. [8]. Vastly different values detected for individual alcohols in volatile profiles of analyzed pear spirits are therefore most likely a consequence of different pear cultivars used as a source of fruit for the production of spirits. Budaljača spirit was characterized by the highest share of alcohols in aromatic profile, while Kalićanka by the smallest (Figure 1). The most abundant alcohol in the investigated pear spirits was phenylethyl alcohol, similarly to what has been reported by several previous studies [8,65]. Phenylethyl is one of the most important flavor alcohols, which, in its pure form, is responsible for rose-like odor. It is quite exclusively a product of the fermentative process [38] and is obtained from L-phenylalanine during fermentation. In higher concentration, it can have negative effects on spirits flavor. The other abundant alcohols in the analyzed pear spirits include 1-hexanol and 1-octanol, and benzyl alcohol (Table 2).

The linalool oxides share in the VOC profile of spirits obtained from old pear cultivars was very low (Figure 1), but they do represent important aroma compounds thanks to their low aroma threshold. Terpenes originate from the raw material and are intensively formed during fruit processing at high temperature and low pH [48]. In such conditions, the release of bound terpenes into free terpenes occurs. The concentration of terpenes has been used to attest the sensorial quality of wines, beers, and distilled beverages [52]. Budaljača cultivar is significantly richer in *cis*- and *trans*-linalool oxides, which allows for the differentiation of Budaljača spirits from others pear spirits.

The presence of phenol compounds in pear spirits is negligible, with the exception of Budaljača pear spirits. This distillate is characterized by a higher content of phenol, 4-ethylphenol, and 4-ethyl-2-methoxyphenol in comparison with other analyzed spirits (Table 2). Interestingly enough, there was no parallel between the content of ethyl lactate and butanedioic acid, diethyl ester on one side and both ethyl phenols on the other side. Very high values for ethyl lactate and butanedioic acid, diethyl ester, and low values for both ethyl phenols were however registered in the Kalićanka pear samples. The opposite result was registered in Budaljača pear samples, where high levels of ethyl phenols and moderate levels of ethyl lactate and butanedioic acid, diethyl ester were found. The relationship between the above-mentioned compounds might be expected because they both represent metabolites of bacterial fermentation. As there was no determined correlation between these compounds, this leads to the conclusion that their content was mandated by the cultivar used in the fermentation process.

Eugenol was the lowest in content, equally across all analyzed pear spirts samples (Table 2). Eugenol is responsible for the clove, medicinal, or phenolic odor, depending on concentration. It has a very low odor threshold, 0.007 µg/L and is mostly connected with positive aroma flavors. Its content has been reported to be higher in pear spirits than in apple spirits [66], which is not in line with the results obtained in this study. Perhaps the higher level of eugenol is a specific characteristic of old apple cultivars.

The overall share of the aldehyde class, within VOCs profile of the analyzed pear spirits, was less than 10% (Figure 1). The most abundant aldehyde was 3-furaldehyde, while benzaldehyde took second place. These results stand in contrast to the findings on apple spirits, where benzaldehyde was the dominant aldehyde while 3-furaldehyde was presents in smaller amounts. The values for the other two aldehydes detected in this study were substantially lower (Table 2).

### 3.4. Sensory Analysis

The results of the sensory evaluations of the nine apple and three pear spirits samples are given in Table 3. All attributes, except fatty/cheesy for pear spirits, were significantly different among spirits produced from different apple and pear cultivars. Overall, the tested spirits were evaluated as having a good quality. The highest score was assigned for odor intensity, typicality, and fruity, followed by floral, spicy, and after taste. Negative attributes like herbaceous, chemical, fatty/cheesy, and pungent were scored as weak or moderate for the majority of spirit samples.

As presented in Table 3, the spirit samples from P. Zelenika apple cultivar had the highest scores for odor intensity, typical apple aroma, as well as for the fruity aroma and after taste. Those attributes were also highly scored for the Bobovec, Masnjača, and Žuja apple spirits samples. The most floral and spicy spirits were those obtained from Samoniklica, Bobovec, Žuja, and Masnjača, i.e., samples with high content of terpenes (Figure 1). Samoniklica apple spirit, which was characterized with highest levels of 4-ethylphenol and 4-ethyl-2-methoxyphenol, was perceived as floral and chemical. The 4-ethylphenol has previously been reported as responsible for varnish, chemical, or pharmacy odor, while 4-ethyl-2-methoxyphenol is responsible for floral, hyacinth odor [57]. G. Delicious and Šarenika apple spirits were judged quite equally in regards to positive sensory attributes, but unlike G. Delicious, Šarenika apple spirits also had high scores for negative sensory attributes such as fatty/cheesy, chemical, and pungent. According to the results of the chemical analyses (Table 2), Šarenika spirts had the highest content of ethyl lactate and butanoic acid, 2-methyl- and 3-methyl. All of these compounds are responsible for strong pungent, cheesy odor. Sarija and Ljepocvjetka apple spirits were similarly described as Šaranika cutivars spirts were, in regards to pungent, fatty, or cheesy aroma.

Pear spirits were perceived as good, i.e., they received high scores for positive sensory attributes and low scores for negative sensory attributes. Budaljača and Krakača pear spirits had better sensory attributes then Kalićanka pear cultivar spirits did.

### 3.5. Principal Component Analysis

**Figure 2:**
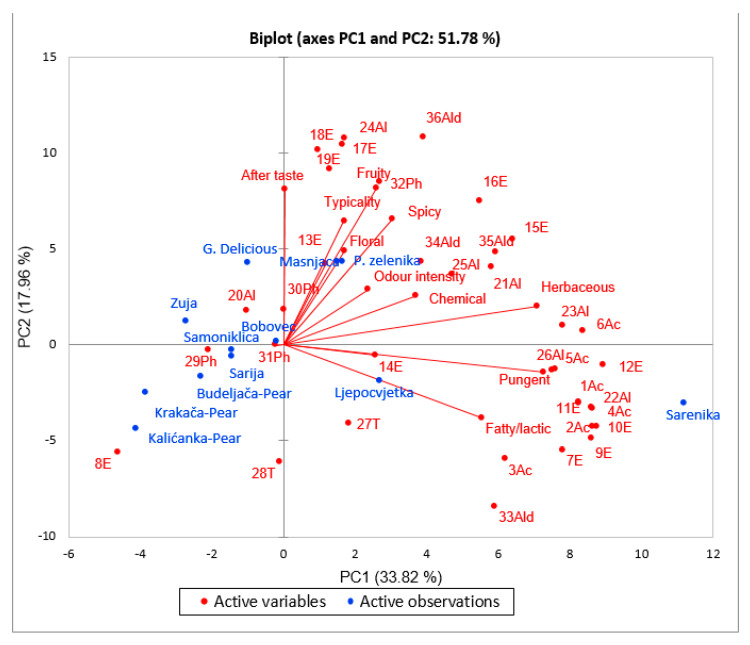
PCA bi-plot for volatile compounds and sensory attributes for 9 apple and 3 pear spirits. 1Ac–Butanoic acid-; 2Ac–Butanoic acid, 3-methyl-; 3Ac–Butanoic acid, 2-methyl-; 4Ac–Hexanoic acid; 5Ac–Octanoic acid; 6Ac–Decanoic acid <n->;7E–Isobutyl acetate; 8E–Lactate <ethyl->; 9E–Isovalerate <ethyl->; 10E–Isoamyl acetate; 11E–Acetate < 2-methylbutyl->, 12E–Hexanoic acid, ethyl ester; 13E–Methoxyacetic acid, 3-methylbutyl ester; 14E–Butanedioic acid, diethyl ester; 15E–Octanoic acid, ethyl ester; 16E–Decanoic acid, ethyl ester; 17E–Dodecanoic acid, ethyl ester; 18E–Tetradecanoate <ethyl->; 19E–Hexadecanoic acid, ethyl ester; 20Al–1-Pentanol; 21Al–1-Hexanol; 22Al–1-Octanol; 23Al–1-Decanol; 24Al–1-Dodecanol; 25Al–Phenylethyl Alcohol; 26Al–Benzyl alcohol; 27T–Linalool oxide <cis->; 28T–trans-Linalool oxide (furanoid); 29Ph–Phenol; 30Ph–Phenol, 4-ethyl-; 31Ph–Phenol, 4-ethyl-2-methoxy-; 32Ph–Eugenol; 33Ald–3-Furaldehyde; 34Ald–5-Methyl-furfural; 35Ald–Benzaldehyde; 36Ald–Nonanal.

Principal component analysis (PCA) was conducted in order to investigate the relationships between the VOCs and sensory attributes of the 12 spirits obtained from eight apple and three pear cultivars. Figure 2 shows the bi-plot for first two principal components. The large differences in VOC contents among spirit samples obtained from different apple cultivars had an effect on the perception of certain aroma notes in the derived spirits.

As presented in Figure 2, there was a very clear differentiation among the apple and pear spirits whereby ethyl lactate and partly *trans*-linalool oxide (furanoid) and phenol managed to differentiate pear spirits from other samples. Pear spirits from Budaljača, Krakača, and Kalićanka cultivars are generally much poorer in VOC content. Among apple spirits, the analyzed samples scattered into two groups. Far removed from these two groups is the spirit obtained from Šarenika apple cultivar. It is very clear that Šarenika apple spirit is characterized by a wealth of chemical compounds, which distinguish this spirit from all other apple spirits. However, the richness of the chemical compounds is not associated with a pleasant aroma. Šarenika apple spirit was characterized by high level of acids. In fact, all detected acids were specific for this spirit. Apart from acids, Šarenika apple spirit also contained many esters (isobutyl acetate, ethyl isovalerate, isoamyl acetate, 2-methylbutyl acetate, and ethyl lactate on the negative side of PC1), alcohols (octanol, decanol, and benzyl alcohol), terpenes (cis- and trans-linalool oxide), and finally 3-furaldehyde. Although isobutyl acetate, ethyl isovalerate, isoamyl acetate, 2-methylbutyl acetate contribute to fruity type of odor and fruity type of flavors, and cis- and trans-linalool oxide are associated with floral odor, Šarenika apple spirit possessed an intense aroma of fatty/cheesy, pungent, and herbaceous. Those negative attributes were due to the high content of short and medium fatty acids that managed to mask the fruit and floral notes. Pungent and herbaceous notes were highly correlated with benzyl alcohol, decanol, octanoic, and decanoic acids, while fatty/cheesy attributes were highly correlated with 3-methyl butanoic acid and 2-methyl butanoic acid.

The first PCA group included spirits derived from P. zelenika, Masnjača, and G. Delicious fruits. Those samples were mainly characterized by pleasant fruity, floral, spicy, typical, and after taste attributes. P. zelenika and Masnjača apple spirits contained overall higher levels of VOCs compared with G. Delicious. The esters ethyl dodecanoate, ethyl tetradecanoate, ethyl hexadecanoate, and alcohol dodecanol were responsible for the typical and fruity attributes and for after taste. Ethyl esters belonging to long-chain fatty acids were correlated to after taste. Those esters have previously been described as providing waxy, candle-like, vegetable oil sensations [67], but in this study they influenced after taste more than ethyl esters of short- or medium-chain fatty acids did. The content of nonanal and eugenol was primarily associated with fruity and floral scents and then also with spicy scent. Spicy note was enhanced by ethyl decanoate. The attributes odor intensity and chemical were in weak correlation with aldehydes methyl-furfural and benzaldehyde, as well as alcohols phenylethyl and hexanol.

The second group consists of spirits that were not clearly defined either by their VOCs or by their chemical properties. Bobovec, Samoniklica, Sarija, and Žuja apple cultivars were characterized by pentanol, while Ljepocvjetka was determined by level of diethyl ester of butanedioic acid and cis-linalool oxide. In this group, Žuja apple spirit could be separated because this sample was very similar in VOCs and sensory scores to G. Delicius spirits (Figure 1 and Table 3). Interestingly enough, phenols, 4-ethyl phenol and 4-ethyl-2-methoxy phenol, content, which was more specific for Bobovec, Samoniklica, Sarija, and Žuja apple spirits, did not negatively impact the sensory properties of these spirits. The presence of any compounds that originate from some defect results affected negatively the scores among sensory attributes. The hypothesis that sensory perception is influenced by the chemical profile is true, but the results of this investigation show that human perception is much more complex than a mere experience of the content of the individual chemical components.

In regards to the loading of the sensory variables, the PC1 was positively correlated with mostly negative sensory attributes such as fatty/cheesy, pungent, herbaceous, and chemical. The PC2 was on the other hand more correlated with positive attributes such as fruity, floral, spicy, and typicality. After taste was exclusively explained by PC2, while odor intensity was connected with both PCs.

## 4. Conclusions

The spirits obtained from apple cultivars Prijedorska zelenika and Masnjača showed intensive fruity and floral aroma, which is more pronounced than in Golden Delicious. The cultivar Žuja was very similar to Golden Delicious in terms of chemical composition and sensory quality. Spirit made from Šarenika apple was characterized by very high level of minor volatile acids and easily distinguishable from all other examined apple spirits. Šarenika is a very specific and promising apple cultivar, but it requires more congeners cleaning during distillations. Samoniklica apple cultivar is characterized by a high level of terpenes, which are varietal compounds and make Samoniklica desirable for flavoring. The old apples cultivars Ljepocvjetka, Bobovec, and Sarija are not usable for the production of spirits due to poor aromatic contributions. The pear cultivar Budaljača proved to be a high-grade aromatic raw material for the production of spirits with an added value.

## Figures and Tables

**Table 1:** Date of fruits delivery to the pilot plant and quality parameters of mashes obtained from old apple and pear cultivars.

Apple Cultivars				Pear Cultivars			
	Date (2014)	pH	Brix (°)		Date (2014)	pH	Brix (°)
Šarenika	31.7.	3.9	13.0				
Masnjača	16.9.	4.3	12.5				
Zuja	20.9.	3.9	15.5				
Lijepocvjetka	20.9.	3.4	12.3	Budaljača	10.10.	3.8	16.3
Bobovec	25.9.	3.8	12.0	Krakača	12.10.	3.9	17.2
P. zelenika	5.10.	3.5	14.8	Kalićanka	12.10.	4.3	12.1
G. Delicious	5.10.	4.2	12.8				
Samoniklica	10.10.	3.4	12.4				
Sarija	14.10.	4.2	16.8				

**Table 2:** Relative amounts of VOCs and coeffcient of variation (%) determined in spirit distilled from old apples (1–8), G. Delicious (9), and old pears (10–12), expressed as average peak areas obtained by HS-SPME-GC-MS (*n* = 4).

	**1Ac**	**2Ac**	**3Ac**	**4Ac**	**5Ac**	**6Ac**	**1Es**	**2Es**	**3 Es**	**4 Es**	**5 Es**	**6 Es**
Ret Index	774	834	847	970	1165	1357	772	815	852	875	878	996
Ion	60	60	74	60	60	60	56	45	88	70	70	88
RT	9.779	11.220	11.474	14.477	18.655	22.253	9.712	10.763	11.694	12.255	12.320	15.088
1. Sarija	270,854	1,897,864	1,134,097	5,440,466	11,540,403	5,473,106	20,148	25,615,398	77,822	246,968	54,213	1,137,197
Coeff. Var%	13.6	15.0	12.2	9.9	11.6	18.6	13.5	8.2	4.7	4.9	6.7	2.4
2. Žuja	404,745	958,421	721,876	4,268,814	4,716,506	2,206,747	10,972	10,526,193	12,858	143,428	29,858	470,427
Coeff. Var%	20.0	13.1	9.8	18.5	25.0	30.0	22.9	7.2	4.5	4.5	5.7	7.2
3. Samoniklica	778,590	11,718,369	3,783,375	7,430,411	11,436,551	3,856,996	5849	13,071,247	139,478	81,766	16,055	665,213
Coeff. Var%	11.9	8.1	7.5	30.9	1.5	4.5	7.2	10.0	4.3	3.8	3.6	0.7
4. P. Zelenika	537,629	5,212,274	1,850,316	9,116,822	19,889,789	6,354,432	12,158	15,311,531	117,007	206,688	44,817	1,450,528
Coeff. Var%	16.1	14.9	12.2	17.3	20.5	40.0	13.0	8.1	11.2	9.4	7.3	7.0
5. Bobovec	1,701,699	13,348,871	15,499,960	11,053,877	9,200,853	3,649,013	32,083	10,073,566	248,489	253,759	72,124	630,296
Coeff. Var%	1.2	1.0	0.7	2.7	8.2	15.8	19.5	0.7	9.5	9.9	10.0	8.0
6. Masnjača	890,200	6,519,060	2,643,604	9,283,436	11,500,953	2,540,646	19,227	10,480,518	89,259	348,854	85,805	1,479,274
Coeff. Var%	5.4	4.5	3.8	10.7	9.4	15.8	8.8	4.0	6.9	8.4	5.9	4.8
7. Ljepocvjetka	1,085,671	24,897,159	8,097,430	14,981,705	18,019,383	6,981,780	109,454	8,198,169	638,285	777,415	206,113	1,020,341
Coeff. Var%	11.3	7.1	6.0	4.7	9.1	20.1	8.0	8.7	5.5	6.3	2.9	3.3
8. Šarenika	3,345,592	64,034,884	19,177,425	35,790,276	23,541,380	7,920,274	138,285	7,867,781	1,888,566	963,543	418,449	3,954,328
Coeff. Var%	5.4	5.0	5.1	3.7	2.2	4.6	2.4	7.5	5.2	3.1	1.9	2.2
9. G. Delicious	468,252	3,731,921	1,527,396	6,877,178	9,325,008	3,357,029	19,530	4,699,610	25,934	318,192	65,980	728,519
Coeff. Var%	13.4	10.5	8.1	14.7	24.7	40.4	11.4	4.6	6.8	6.2	5.2	5.7
10. Budeljača	1,054,843	4,087,594	1,933,705	15,243,822	8,345,600	2,954,199	27,923	18,255,059	70,480	166,075	76,796	857,675
Coeff. Var%	24.7	25.7	23.1	12.4	9.3	8.5	23.3	2.6	24.6	22.9	21.9	19.8
11. Krakača	239,948	2,877,129	6,474,659	3,680,745	5,953,638	2,447,567	24,551	15,732,250	139,528	225,167	56,366	434,219
Coeff. Var%	17.9	17.6	25.3	19.6	20.0	38.2	8.9	4.5	7.8	3.8	3.1	2.2
12. Kalićanka	209,055	802,676	5,879,745	3,206,562	6,585,451	2,316,784	17,029	26,325,508	27,097	145,272	34,007	404,601
Coeff. Var%	3.5	3.5	4.2	3.9	4.0	7.45	15.4	9.6	11.2	9.95	7.6	6.7
	**7 Es**	**8 Es**	**9 Es**	**10 Es**	**11 Es**	**12 Es**	**13 Es**	**1Alc**	**2Alc**	**3Alc**	**4Alc**	**5Alc**
Ret Index	1074	1175	1195	1394	1593	1793	1994	765	868	1069	1273	1477
Ion	45	101	88	88	88	88	88	55	56	56	70	55
RT	16.764	18.827	19.220	22.848	26.069	28.962	31.583	9.539	12.064	16.669	20.704	24.237
1. Sarija	256,022	980,484	4,193,961	4,899,822	935,126	70,377	151,243	123,298	9,548,159	401,853	190,239	72,382
Coeff. Var%	7.0	6.4	4.6	5.8	10.3	21.3	28.7	7.7	6.8	4.3	13.5	68.5
2. Žuja	257,937	1,588,658	1,171,207	2,098,920	531,862	88,457	127,524	185,326	14,297,598	824,466	169,113	48,693
Coeff. Var%	5.4	4.0	12.0	13.9	8.4	44.6	47.9	8.5	5.0	1.9	8.8	36.9
3. Samoniklica	102,546	1,742,331	2,070,096	1,708,790	242,206	48,764	107,995	123,354	9,531,321	456,679	104,176	37,258
Coeff. Var%	5.4	2.1	6.7	13.2	18.8	21.5	16.2	5.4	5.0	0.5	10.0	37.7
4. P. Zelenika	299,448	1,985,593	5,825,153	5,782,441	1,202,876	88,652	177,679	154,058	16,489,171	3,169,370	694,344	77,496
Coeff. Var%	4.1	1.2	5.4	7.6	14.5	20.9	7.2	7.8	6.1	4.1	2.9	17.6
5. Bobovec	158,171	1,583,726	991,492	1,316,029	158,193	25,046	77,600	203,747	13,584,232	906,906	124,877	52,869
Coeff. Var%	4.1	3.2	11.1	4.8	7.6	28.6	29.9	9.7	7.7	7.1	21.6	47.8
6. Masnjača	326,140	1,471,636	4,841,737	5,180,169	2,104,683	220,928	342,550	235,132	15,290,639	720,034	187,591	62,040
Coeff. Var%	9.5	1.8	4.1	2.8	6.5	9.4	17.1	10.3	6.7	2.7	7.5	23.8
7. Ljepocvjetka	198,718	1,014,310	2,466,074	2,930,255	466,219	63,438	131,641	92,874	7,833,311	2,846,081	426,088	32,088
Coeff. Var%	2.7	2.4	7.1	9.3	10.9	24.3	26.4	25.4	4.0	2.2	4.9	18.8
8. Šarenika	252,108	1,959,444	5,196,883	4,744,525	568,457	52,844	155,490	131,383	20,199,936	9,044,005	851,032	43,552
Coeff. Var%	5.5	2.3	1.1	2.2	4.0	8.3	16.1	29.3	5.6	3.2	11.9	55.8
9. G. Delicious	284,419	925,068	2,377,616	3,904,078	1,903,381	218,142	544,974	128,124	9,867,953	540,392	377,357	99,194
Coeff. Var%	3.9	4.1	9.7	12.4	16.6	16.1	12.7	5.4	3.5	2.5	2.5	12.5
10. Budeljača	377,907	874,095	725,121	871,105	180,606	21,785	63,893	442,893	17,500,634	1,545,211	254,715	29,253
Coeff. Var%	11.3	13.8	25.7	33.3	48.8	84.2	88.9	11.9	5.1	13.9	5.3	69.9
11. Krakača	98,705	420,906	1,105,219	1,340,998	238,070	23,583	65,834	115,630	6,520,646	546,273	123,311	18,328
Coeff. Var%	6.5	31.8	3.3	12.1	34.2	67.3	87	5.3	4.1	1.6	5.3	56.6
12. Kalićanka	263,715	2,421,734	1,025,579	843,896	240,286	36,012	85,673	78,250	4,775,326	892,434	184,128	12,429
Coeff. Var%	3.75	0.6	5.35	2.55	1.5	4.8	26.35	12.7	4.2	4.4	6.1	26
	**6Alc**	**7Alc**	**1T**	**2T**	**3T**	**4T**	**5T**	**6T**	**1Ald**	**2Ald**	**3Ald**	**4Ald**
Ret Index	1127	1041	1083	1099	977	1168	1294	1375	835	968	971	1107
Ion	91	79	59	59	94	107	137	164	96	110	106	57
RT	17.858	16.069	16.964	17.290	14.638	18.681	21.093	22.516	11.269	14.435	14.507	17.453
1. Sarija	13,059,356	409,043	120,321	83,613	46,121	61,098	3364	56,235	3,727,210	121,330	5,123,242	169,313
Coeff. Var%	3.7	5.8	7.3	7.9	12.3	102.7	70.4	26.4	10.3	23.4	6.3	8.0
2. Žuja	13,132,246	474,151	414,447	305,385	141,906	109,745	9875	33,154	1,604,679	41,420	6,125,782	200,269
Coeff. Var%	5.8	3.0	2.9	3.2	58.0	29.4	17.0	8.4	4.4	3.5	3.4	18.0
3. Samoniklica	13,141,421	1,189,982	861,447	606,744	320,981	11,026,582	1,508,903	93,262	2,969,075	69,348	4,419,921	123,492
Coeff. Var%	3.3	4.7	3.9	4.3	4.6	3.7	2.0	4.7	4.9	6.5	1.8	9.3
4. P. Zelenika	11,296,844	440,944	271,195	201,091	93,557	4,363,149	178,046	1,151,391	1,171,497	35,059	5,562,958	231,384
Coeff. Var%	1.4	1.6	5.9	5.8	2.3	12.8	0.7	1.8	5.0	7.3	4.3	6.8
5. Bobovec	14,799,501	693,343	316,102	247,632	106,496	361,903	40,799	283,992	3,971,465	515,541	3,435,460	153,332
Coeff. Var%	4.5	7.9	3.2	3.0	3.0	4.2	6.9	5.8	6.0	4.9	8.1	7.4
6. Masnjača	25,026,431	1,929,611	258,464	136,467	76,992	302,128	22,402	442,601	4,015,113	337,256	8,588,380	245,259
Coeff. Var%	0.5	3.5	4.9	5.1	5.0	7.4	5.3	0.9	4.9	3.7	4.0	7.2
7. Ljepocvjetka	20,896,390	890,413	238,843	188,818	86,757	634,715	22,945	39,369	5,013,573	96,761	1,729,915	156,940
Coeff. Var%	2.1	2.3	2.8	2.9	3.6	1.7	2.4	3.9	4.7	4.5	2.5	48.1
8. Šarenika	19,150,146	2,549,366	826,473	568,913	80,404	758,497	139,508	184,723	9,000,836	402,567	8,681,267	191,734
Coeff. Var%	3.3	2.0	4.3	4.7	5.0	2.2	1.0	0.8	4.5	2.9	2.1	10.4
9. G. Delicious	19,925,204	332,312	190,491	170,661	101,836	438,162	14,742	131,890	2,674,325	563,915	1,686,725	299,807
Coeff. Var%	6.6	11.5	2.8	2.2	6.6	6.8	2.4	2.8	3.7	2.6	3.7	3.6
10. Budeljača	14,012,827	704,510	1,213,702	1,019,895	204,919	183,886	36,840	19,507	3,193,477	56,377	1,062,875	101,125
Coeff. Var%	8.6	15.6	9.2	8.7	41.1	32.6	26.1	34.0	16.8	23.7	34.2	19.8
11. Krakača	11,439,001	480,656	274,050	398,446	85,658	56,022	13,583	21,194	4,644,915	69,719	1,136,044	48,390
Coeff. Var%	2.8	5.4	3.5	3.2	6.3	7.3	3.9	25.1	4.9	9.5	41	3.2
12. Kalićanka	13,651,276	536,944	271,454	391,803	77,812	56,422	2890	20,241	5,316,681	49,290	764,781	38,783
Coeff. Var%	3.5	3.05	1.8	1.6	12.2	4.45	3.2	9.45	5.65	3.0	17.55	10.4

ACIDS: 1Ac–Butanoic acid-; 2Ac–Butanoic acid, 3-methyl-; 3Ac–Butanoic acid, 2-methyl-; 4Ac–Hexanoic acid; 5Ac–Octanoic acid; 6Ac–Decanoic acid <n->; ESTERS 1Es–Isobutyl acetate; 2Es–Lactate <ethyl->; 3Es–Isovalerate <ethyl->; 4Es–Isoamyl acetate; 5Es–Acetate <2-methylbutyl->, 6Es–Hexanoic acid, ethyl ester. ESTARS: 7Es–Methoxyacetic acid, 3-methylbutyl ester; 8Es–Butanedioic acid, diethyl ester; 9Es–Octanoic acid, ethyl ester; 10Es–Decanoic acid, ethyl ester; 11Es–Dodecanoic acid, ethyl ester; 12Es–Tetradecanoate <ethyl->; 13Es–Hexadecanoic acid, ethyl ester; ALCOHOLS: 1Alc–1-Pentanol; 2Alc–1-Hexanol; 3Alc–1-Octanol; 4Alc–1-Decanol; 5Alc–1-Dodecanol. ALCOHOLS: 6Alc–Phenylethyl Alcohol; 7Alc–Benzyl alcohol; TERPENES: 1T–Linalool oxide <cis->; 2T–trans Linalool oxide (furanoid); 3T–Phenol; 4T–Phenol, 4-ethyl-; 5T–Phenol, 4-ethyl-2-methoxy-; 6T–Eugenol; ALDEHYDES: 1Ald–3-Furaldehyde; 2Ald–5-Methyl-furfural; 3Ald–Benzaldehyde; 4Ald–Nonanal.

**Table 3:** Results of sensory evaluations of the spirits obtained from old apple and pear cultivars in B&H.

Sensory Attributes	Spirits from Apple Cultivars									Spirits from Pear Cultivars		
	Sarija	Zuja	Samoniklica	P. zelenika	Bobovec	Masnjača	Lijepocvjetka	Šarenika	G.Delicious	Budaljača	Krakača	Kalićanka
Odor intensity	2.57 e	4.05 c	4.10 cd	4.83 a	4.73 a	3.85 cd	3.48 d	4.58 ab	4.23 bc	4.43 a	4.33 a	3.55 b
Typicality	3.28 d	4.65 ab	4.5 ab	4.93 a	4.63 ab	3.98 c	3.53 c	4.38 bc	4.38 bc	4.28 a	4.23 a	3.13 b
Fruity	3.63 b	4.10 b	3.89 b	4.75 a	4.33 ab	4.68 a	4.05 b	3.93 b	4.10 b	4.53 a	3.53 b	2.95 b
Floral	1.53 d	3.73 ab	4.25 a	3.48 bc	4.03 ab	3.75 ab	3.05 c	3.38 bc	3.23 bc	3.75 a	3.05 a	1.93 b
Herbaceous	0.78 b	1.05 b	3.03 a	2.68 a	3.00 a	3.38 a	2.90 a	3.75 a	1.18 b	1.38 a	0.73 b	1.18 a
Spicy	1.90 c	3.15 ab	3.70 a	3.70 a	3.90 a	3.58 a	3.18 a	2.95 ab	2.48 bc	2.13 a	2.93 b	1.38 c
Chemical	3.48 a	2.10 bc	3.50 a	2.48 ab	1.60 c	3.00 ab	2.40 abc	2.85 ab	1.73 c	0.95 a	1.38 b	1.68 b
Fatty/cheesy	3.85 a	1.95 c	1.70 c	2.05 c	1.33 c	1.73 c	2.9 b	3.35 ab	1.43 c	1.65 ns	1.9 ns	1.65 ns
Pungent	2.30 b	1.80 c	2.20 b	2.50 ab	2.00 bc	1.80 c	3.00 a	3.10 a	1.80 c	1.50 b	1.30 a	1.90 c
After taste	3.20 d	4.15 ab	4.28 ab	4.65 a	4.50 ab	3.93 bc	3.8 bcd	3.33 cd	3.85 bcd	3.90 a	3.78 a	2.60 b

Different letters in the same row, separately for the fruit species, denote significant differences (*p* < 0.05). Scores shown are mean values of the sensory panel using 5-point scale: 1—very weak, 2—weak, 3—moderate, 4—strong, and 5—very strong. Standard deviation ≤ 0.75.

## Data Availability

All data are presented in this manuscript.
